# Drug-specific in vitro release of IFN-gamma in patients with delayed cutaneuos drug hypersensitivity reactions

**DOI:** 10.1186/2045-7022-5-S1-O3

**Published:** 2015-03-11

**Authors:** Grzegorz Porebski, Magdalena Bosak

**Affiliations:** 1 Jagiellonian University, Dep. of Clinical and Environmental Allergology, Krakow; Poland; 2 Jagiellonian University, Department of Neurology, Krakow, Poland

## Background

One of the challenges for management of drug hypersensitivity reactions (DHR) is to detect a culprit drug. Although measures of interferon IFN-gamma production by patients' drug-specific T cells have been the widely utilized cytokine assay, a systematic comparison of different methods has not yet been reported.

## Method

A total of 16 patients with clinically well-established maculopapular exanthema due to antiepileptic drugs hypersensitivity in remission state and 15 drug-exposed control donors without DHR were included to the study. Peripheral blood mononuclear cells of investigated individuals were isolated and cultured under defined conditions with drugs. IFN-gamma production was measured with electrochemiluminescence array assay and ELISA (cytokine level in cell culture supernatant), ELISpot (cytokine secreting cells), flow cytometry (intracellular staining in CD3+ CD4+ cells).

## Results

IFN-gamma production could be demonstrated in 13 of 16 patients using electrochemiluminescence assay (sensitivity 81%), in 8 of 16 patients using ELISA (sensitivity 50%), in 6 of 16 and 7 of 16 patients using ELISpot (sensitivity 46%) and flow cytometry (sensitivity 57%), respectively. The sensitivity of combined measurements of drug-specific IFN-gamma by ELISpot, ELISA and flow cytometry achieved 88%. Healthy controls showed negative drug-specific IFN-gamma production in contrast to individuals with a known sensitivity in all tested read-out systems. The assays demonstrated a test specificity of 100% (electrochemiluminescence), 93% (ELISA), 100% (ELISpot) and 100% (flow cytometry).

## Conclusion

Analysis of drug-specific IFN-gamma production by means of different assays proved a useful and reliable approach for the in vitro detection of drug hypersensitivities in the investigated population. Electrochemiluminescence array assay offers distinct advantage over the other tested assays, including a greater sensitivity, but its availability is limited because of the costs. Also combining different assays may be a feasible approach to identify the causative drug of DHR.

